# The regulatory role of NAAG-mGluR3 signaling on cortical synaptic plasticity after hypoxic ischemia

**DOI:** 10.1186/s12964-022-00866-8

**Published:** 2022-04-20

**Authors:** Kexin Li, Meng Lu, Mengxu Cui, Xiaoming Wang, Yang Zheng

**Affiliations:** Department of Radiology, Shengjing Hospital of China Medical University, No. 36, Sanhao Street, Heping District, Shenyang, 110004 People’s Republic of China

**Keywords:** Hypoxic-ischemic injury, N-acetylaspartylglutamate, Metabotropic glutamate receptor, Synaptic plasticity

## Abstract

**Background:**

Synapses can adapt to changes in the intracerebral microenvironment by regulation of presynaptic neurotransmitter release and postsynaptic neurotransmitter receptor expression following hypoxic ischemia (HI) injury. The peptide neurotransmitter N-acetylaspartylglutamate (NAAG) exerts a protective effect on neurons after HI and may be involved in maintaining the function of synaptic networks. In this study, we investigated the changes in the expression of NAAG, glutamic acid (Glu) and metabotropic glutamate receptors (mGluRs), as well as the dynamic regulation of neurotransmitters in the brain after HI, and assessed their effects on synaptic plasticity of the cerebral cortex.

**Methods:**

Thirty-six Yorkshire newborn pigs (3-day-old, males, 1.0–1.5 kg) were selected and randomly divided into normal saline (NS) group (n = 18) and glutamate carboxypeptidase II inhibition group (n = 18), both groups were divided into control group, 0–6 h, 6–12 h, 12–24 h, 24–48 h and 48–72 h groups (all n = 3) according to different post-HI time. The content of Glu and NAAG after HI injury were detected by 1H-MRS scanning, immunofluorescence staining of mGluRs, synaptophysin (syph) along with postsynaptic density protein-95 (PSD95) and transmission electron microscopy were performed. ANOVA, Tukey and LSD test were used to compare the differences in metabolite and protein expression levels among subgroups. Correlation analysis was performed using Pearson analysis with a significance level of α = 0.05.

**Results:**

We observed that the NAAG and mGluR3 expression levels in the brain increased and then decreased after HI and was significantly higher in the 12–24 h (P < 0.05, Tukey test). There was a significant positive correlation between Glu content and the expression of mGluR1/mGluR5 after HI with r = 0.521 (*P* = 0.027) and r = 0.477 (*P* = 0.045), respectively. NAAG content was significantly and positively correlated with the level of mGluR3 expression (r = 0.472, *P* = 0.048). When hydrolysis of NAAG was inhibited, the expression of synaptic protein PSD95 and syph decreased significantly.

**Conclusions:**

After 12–24 h of HI injury, there was a one-time elevation in NAAG levels, which was consistent with the corresponding mGluR3 receptor expression trend; the NAAG maintains cortical synaptic plasticity and neurotransmitter homeostasis by inhibiting presynaptic glutamate vesicle release, regulating postsynaptic density proteins and postsynaptic receptor expression after pathway activation.

**Video abstract**

**Supplementary Information:**

The online version contains supplementary material available at 10.1186/s12964-022-00866-8.

## Background

After hypoxic ischemic brain injury, nerve cells and neural networks undergo adaptive changes to maintain cerebral homeostasis, which are referred to as neuroplasticity. These changes include neural regeneration and changes in synaptic structure and function at the cellular level [1–3]. Synaptic plasticity is an important mechanism for the formation and maintenance of functional neural circuits, which can be dynamically regulated in physiological and pathological states, further causing changes in brain function, behavior, and mental activity [4, 5].

Synaptic activity requires the involvement of a large amount of neuromodulatory substances, including neurotransmitters and neuropeptides. Glutamic acid (Glu) is the most abundant cerebral excitatory neurotransmitter [6]. Excitotoxic effect caused by Glu accumulation in the synaptic gap is one of the main causes of neuronal cell damage after hypoxic ischemia (HI), as increased Glu release promotes glutamate receptor overactivation and intracellular calcium overload hierarchical damage response [7]. N-acetylaspartylglutamate (NAAG) is a kind of peptide neurotransmitter, and its main physiological function is to participate in amino acid metabolism in the nervous system as well as to regulate neurotransmitter release and thus influence neuroplasticity [8, 9]. Several studies have indicated that NAAG can attenuate excitotoxicity caused by increased Glu transmission and hence exert neuroprotective effects [10–12], which is determined by the metabolic processes and signaling patterns of NAAG in the brain. NAAG synthetase (NAAGS) catalyzes the biosynthesis of NAAG from N-acetyl-aspartate (NAA) and Glu (NAAGS) [13, 14] through a mechanism that consumes Glu and helps to minimize the excitotoxic effects of Glu accumulation in pathological situations, hence reducing neuronal damage.

NAAG can also reduce excitotoxicity by affecting Glu binding to the metabolic glutamate receptor (mGluRs). mGluRs includes three subtypes, type I mGluR (mGluR1,5) with postsynaptic localization and known to regulate neuronal excitability, type II (mGluR2,3) and type III mGluR (mGluR4,6,7,8) with presynaptic localization and known to regulate Glu release [15]. NAAG is released from the postsynapse and competitively binds to type II metabotropic glutamate receptor 3 (mGluR3). Further, this complex acts as a retrograde neurotransmitter and negatively regulates Glu signaling by lowering Glu release and excitotoxicity by inhibiting intra- and extrasynaptic N-methyl-D-aspartic acid receptor (NMDAR) [9, 16–18]. However, NAAG acts for a short time and is quickly hydrolyzed into NAA and Glu by glutamate carboxypeptidase II (GCP-II) [14, 19]. The use of GCP-II inhibitors such as 2-phosphonomethyl pentanedioic acid (2-PMPA) can increase NAAG levels, thus promoting the neuroprotective effect of NAAG [20, 21]. In addition to attenuating neuronal damage caused by Glu over-release, activated NAAG-mGluR3 pathway may maintain Glu homeostasis and regulate postsynaptic responses and plasticity changes across polysynaptic connections in real time [15, 22]. Pinheiro et al. [23] demonstrated that activation of presynaptic mGluRs leads to synaptic inhibition and is involved in the long-term and short-term regulation of synaptic plasticity.

Under action potential evocation, synaptic vesicles (SVs) translocate to the active zone, fuse with the presynaptic plasma membrane, and subsequently release neurotransmitters. Short-term presynaptic plasticity could be related to SVs' ability to fuse and bind to the plasma membrane of the activated zone [24, 25]. Synaptophysin (syph) is a presynaptic terminal marker protein that is widely distributed in presynaptic membrane vesicles and can reflect the density, distribution area, and functional state of the synapse [26]. It is also involved in calcium binding processes, neurotransmitter release, and synaptic vesicle recycling. Syph is associated with synapse formation during CNS development, thus reflecting synaptic plasticity [27, 28]. Postsynaptic density (PSD) is a dense material located at the postsynaptic membrane, mainly composed of receptor, skeletal proteins etc., among which PSD95 is an important skeletal protein maintaining postsynaptic receptor function and is involved in the maintenance of glutamate postsynaptic receptor stabilization and acts as an important indicator of changes in postsynaptic components [29].

In the present study, we investigated the changes in the NAAG, Glu and Glu receptor expression after HI, analyzed the dynamic regulation process of neurotransmitters in the brain after HI injury, and explored the relationship with syph and PSD95 expression. The effect of NAAG-mGluR3 pathway on synaptic plasticity was further investigated.

## Methods

### Experimental animals and HI model

The experimental animals used in this study were 36 newborn Yorkshire pigs 3 days old (body mass 1.0–1.5 kg, males). They were randomly assigned into normal saline (NS) group (n = 18) and 2-PMPA group (n = 18), both groups were divided into control group, 0–6 h, 6–12 h, 12–24 h, 24–48 h and 48–72 h groups (all n = 3) according different post-HI time. The experimental procedures related to experimental animals were approved by the Institutional Committee for Animal Care and Use of our hospital. The establishment of HI model was performed following protocols of previously published studies [30] and Additional file 1. At 30 min after completion of modeling, 2.5 mg/kg 2-PMPA [21] (S0189, Selleck) was intraperitoneally injected in the GCP-II inhibition group, and normal saline (NS) group animals were injected with equal volume of NS.

### ^1^H-MRS scanning

Philips Achieva 3.0 T MRI scanner (Philips Healthcare, The Netherlands) was used for scanning. Gradient coil was used for emission and 8-channel RF coil for receiving. ^1^H-MRS scan sequence and parameters were single voxel PRESS sequence, TR = 2000 ms, TE = 37 ms; NSA = 64; VOI = 10 × 10 × 10 mm. Image were post-processed by LCModel software package.

### Immunofluorescence staining and image analysis

Brain tissues were fixed in formaldehyde for 72 h, dehydrated in gradient ethanol, made transparent in xylene, and embedded in paraffin wax before being cut into 4-μm pathological sections using an automatic microtome (HM340E, Thermo Scientific, MI, USA). Immunofluorescence staining was performed for mGluR3, mGluR1, mGluR5, syph and PSD95. The staining procedure was as follows: following xylene dewaxing and gradient ethanol hydration of the sections, the antigen was recovered by citrate buffer (0.01 M, pH = 6.0) in microwave for 7.5 min over high heat. Normal goat serum was used for blocking of non-specific antibody binding at room temperature for 30 min. Primary antibodies, such as Anti-Metabotropic Glutamate Receptor 1 antibody ab27199; Anti-Metabotropic Glutamate Receptor 5 antibody ab76316; Anti-Metabotropic Glutamate Receptor 3 antibody ab188750; Anti-Synaptophysin antibody ab52636, Anti-PSD95 antibody ab18258 were incubated at 4 °C overnight. The sections were further incubated with secondary antibodies (Alexa Fluor 488-labeled goat anti-rabbit IgG 1:100, Immunoway, RS3211) for 4 h at room temperature. Finally, the sections were incubated with DAPI (4',6-Diamidino-2-Phenylindole, Dihydrochloride, ab104139) for 5 min for nuclear staining.

Immunofluorescence images were acquired (× 200 and × 400) by confocal laser scanning microscope (LSM880, Axio Examiner; ZEISS, Germany). After image acquisition, protein expression was determined using ImageJ software (Java 1.6.0; National Institutes of Health, USA), and the average optical density (OD) value was used to represent the intensity of protein staining.

### Western blot analysis

For protein extraction, 100 mg of cerebral cortical tissue were weighed in each group and homogenized and lysed using 1 ml RIPA and 10 μl PMSF. Further, after centrifugation the supernatant was collected for protein concentration determination followed by denaturation using 5X loading buffer at 100 ℃ for 5 min. SDS-PAGE electrophoresis was performed by loading 30 μg of protein to each lane with electrophoresis conditions at 140 V for 60 min. Further, the sample were transferred to a PVDF membrane and blocked with 5% skim milk for 2 h. The membrane was further incubated with primary antibodies such as GCPII (1:2000, ab133579), syph(1:1000, ab52636), PSD95 (1: 1000, ab18258), β-actin (1: 1000, ab8226), at 4 ℃ overnight and then incubated with horseradish peroxidase (HRP) conjugated rabbit and mouse IgG secondary antibody (1: 10,000, proteintech, SA00001-1; SA00001-2) for 2 h at room temperature.

The membrane was further visualized using the GE Imaging System (Amersham Imager 680; GE, Japan), and quantitatively analysed using the ImageJ software (Java1.6.0; National Institutes of Health).

### Transmission electron microscopy

Fresh cerebral cortical tissue was obtained, fixed in 2.5% glutaraldehyde solution at 4 ℃ for 24 h, post fixed with 1% osmium solution for 2 h, gradient ethanol, acetone dehydrated, epoxy embedded, and cut into 70 nm ultrathin tissue sections. The sections were stained by uranium acetate for 10 min and lead citrate for 5 min, washed in distilled water, and naturally dried. Sections were observed using a transmission electron microscope (TEM, transmission electron microscopy) (JEM-1400Flash, Japan).

### Statistical analysis

The normality test for quantitative data was performed using the Shapiro–Wilk method. One-way ANOVA were used to compare the total differences. Post-hoc multiple tests were performed between multiple groups using the Tukey test, while paired comparison using LSD test, correlation analysis was performed using Pearson analysis, and *P* < 0.05 was considered a statistically significant difference. All statistical analyses were performed using SPSS (version 22.0; IBM, Armonk, New York) and GraphPad Prism (version 8.0.2; GraphPad Software, San Diego, California) software.

## Results

### Changes in cortical mGluR3, mGluR1, and mGluR5 expression after HI injury

**Fig. 1:**
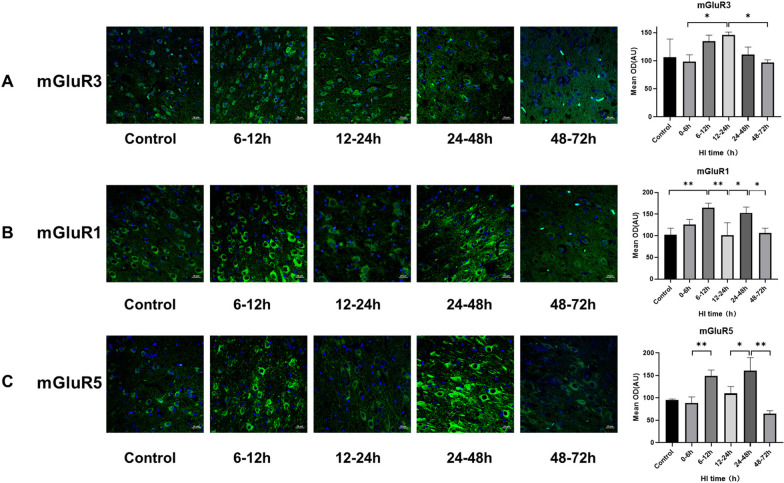
mGluR3, mGluR1, mGluR5 expression changes after HI injury (× 400). **A** Trends of mGluR3 immunostaining in the brain and mean optical density in after-HI groups at 6–12 h, 12–24 h, 24–48 h and 48–72 h, with respective controls. Green fluorescence represents mGluR3. **B** Trends of mGluR1 immunostaining and mean optical density in after HI groups at 6–12 h, 12–24 h, 24–48 h and 48–72 h. Green fluorescence represents mGluR1. **C** Trends of mGluR5 staining and mean optical density at 6–12 h, 12–24 h, 24–48 h and 48–72 h groups after HI, with respective controls. The green fluorescence represents mGluR5. **P < 0.01, *P < 0.05, data expressed as mean ± standard deviation

Initially, we assessed the changes occurring in mGluR levels during different time periods after HI. The mGluR3 expression at 12–24 h was significantly higher than in 0–6 h and 48–72 h post-HI group (*P* = 0.035; 0.027, Tukey test) (Fig. 1A). The mGluR1 expression was significantly increased at 6–12 h than in the control group (*P* = 0.005, Tukey test), decreased at 12–24 h (*P* = 0.005, Tukey test), and increased again at 24–48 h (*P* = 0.021, Tukey test) (Fig. 1B). mGluR5 expression were significantly higher in the 6–12 h than in the 0–6 h group (*P* = 0.006, Tukey test). Post-HI mGluR5 increased at 24–48 h than 12–24 h and 48–72 h group (*P* = 0.018; *P* < 0.001, Tukey test) (Fig. 1C).

### ^1^H-MRS analysis of NAAG and Glu alterations and correlation with metabotropic glutamate receptor changes after HI injury

**Fig. 2:**
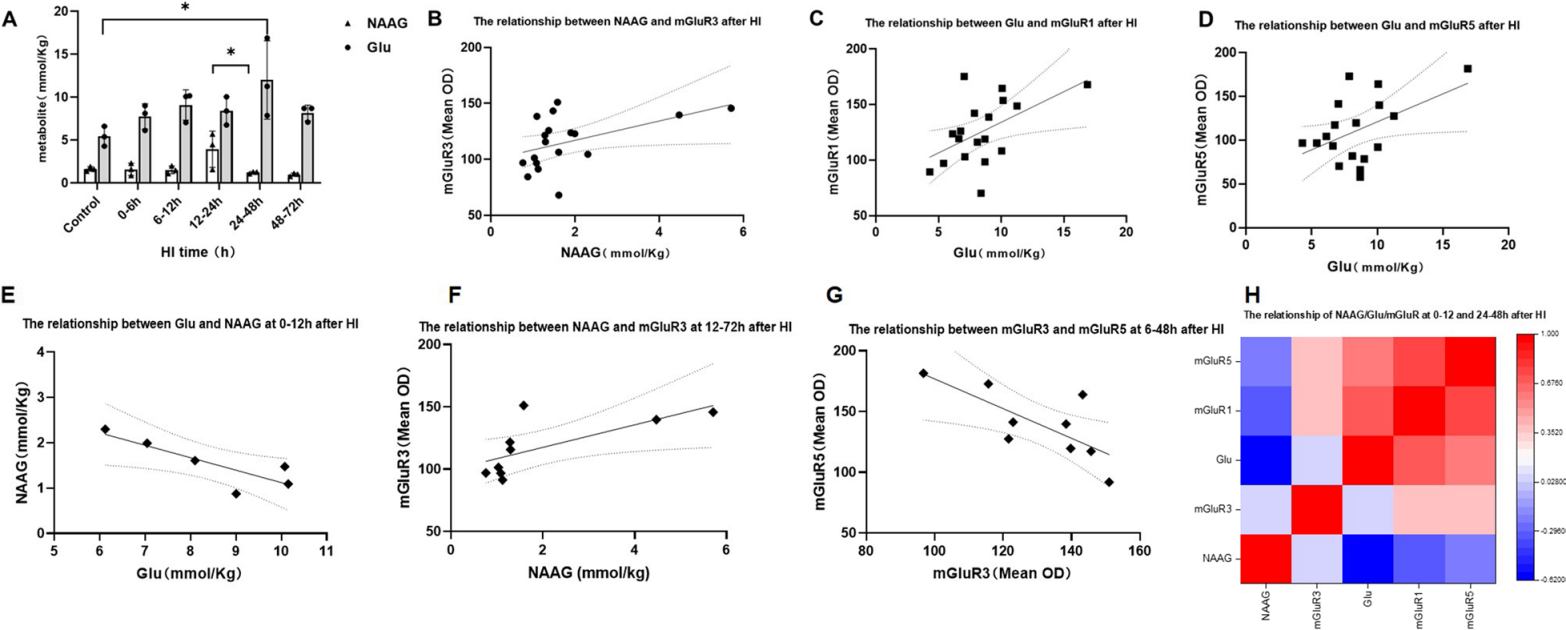
Changes in NAAG and Glu and scatter plots with mGluRs after HI. **A** Changes in NAAG and Glu after HI injury. **B** Scatter plot of the correlation between NAAG and mGluR3 expression after HI. **C** Scatter plot of the correlation between Glu and mGluR1 expression after HI. **D** Scatter plot of the correlation between Glu and mGluR5 expression after HI. **E** Scatter plot of the correlation between Glu and NAAG at 0–12 h after HI. **F** Scatter plot of the correlation between NAAG and mGluR3 expression at 12–72 h after HI. **G** Scatter plot of the correlation between mGluR3 and mGluR5 expression at 6–48 h after HI. **H** Heatmap of NAAG/Glu/mGluR at 0–12 h and 24–48 h after HI (*P < 0.05, data expressed as mean ± standard deviation)

^1^H-MRS was used to evaluate changes in NAAG and Glu in the brain at various time intervals following HI, as indicated in Fig. 2A. NAAG content in the brain at 12–24 h after HI was statistically higher than the group at 24–48 h after HI (*P* = 0.039, Tukey test). The Glu content increased at 24–48 h after HI when compared with the control group (*P* = 0.037, Tukey test). Correlation of NAAG, Glu and metabolic glutamate receptor changes after HI injury is shown in Fig. 2B–H. NAAG content was significantly and positively correlated with the level of mGluR3 expression (r = 0.472, *P* = 0.048) (Fig. 2B), especially at 12–72 h after HI (r = 0.696, *P* = 0.037) (Fig. 2F). We observed a significant positive correlation between Glu content and the expression of mGluR1/mGluR5 after HI with r = 0.521 (*P* = 0.027) and r = 0.477 (*P* = 0.045), respectively (Fig. 2C, D). The correlation heatmap showed that the Glu content were significantly positively correlated with the level of mGluR1/5 (r = 0.692, *P* = 0.013; r = 0.617, *P* = 0.032) at 0–12 h and 24–48 h (Fig. 2H). NAAG and Glu were negatively correlated at 0–12 h after HI (r = -0.838, *P* = 0.037) (Fig. 2E). Besides, mGluR3 and mGluR5 were positively correlated at 6–48 h after HI (r = 0.736, *P* = 0.024) (Fig. 2G).

### Changes in cortical syph and PSD95 expression after HI injury

**Fig. 3:**
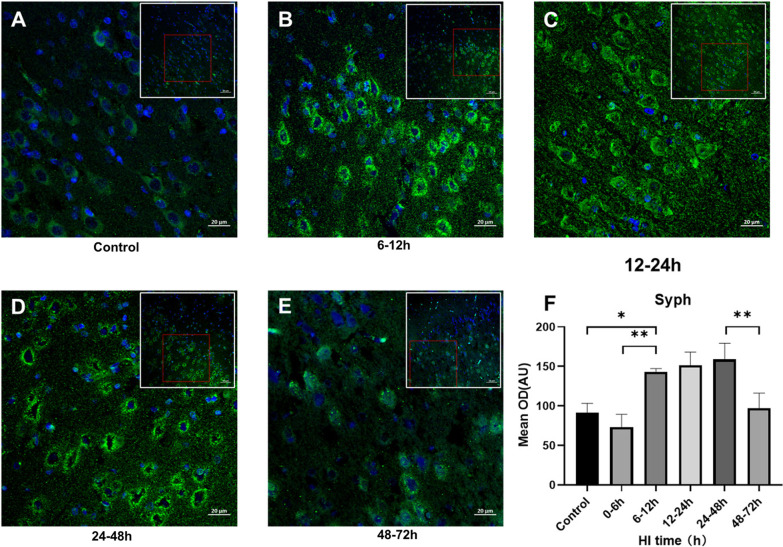
Changes in synaptophysin expression after HI injury (× 200, × 400). **A**–**F**. Syph staining of the brain sections and mean integrated optical density changes in 6–12 h, 12–24 h, 24–48 h and 48–72 h groups after HI, with respective controls. Green fluorescence represents syph. (**P < 0.01, *P < 0.05, data are expressed as mean ± standard deviation)

**Fig. 4:**
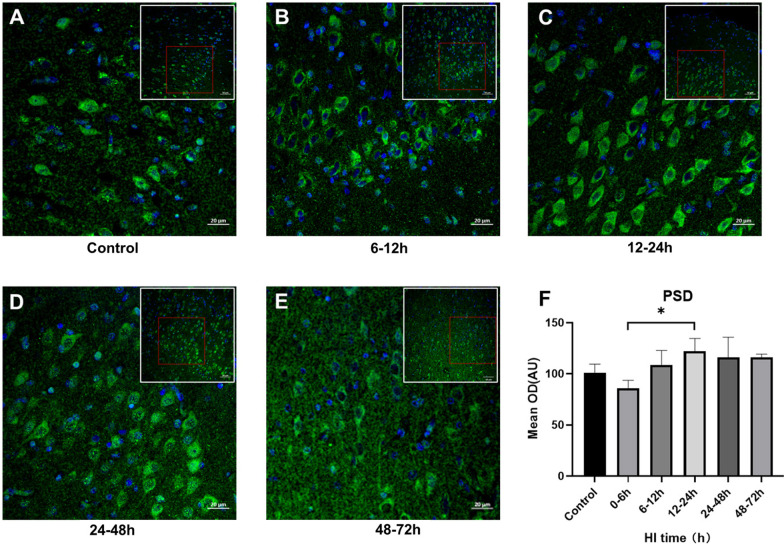
Changes in PSD95 expression after HI injury (× 200, × 400). **A**–**E** PSD95 staining of the brain sections and mean integrated optical density changes in 6–12 h, 12–24 h, 24–48 h and 48–72 h groups after HI, with respective controls. Green fluorescence represents PSD95. **F** The PSD95 fluorescence intensity increased and then decreased after HI, increased at 6–12 h, lasted until 24–48 h and significantly decreased at 48–72 h. (**P < 0.01, *P < 0.05, data are expressed as mean ± standard deviation)

Next, we studied the changes in cortical syph and PSD95 expression after HI injury. As compared to the control group, syph was significantly increased 6–12 h after HI (*P* = 0.016, Tukey's test) and lasted until 24–48 h, then significantly decreased at 48–72 h (*P* = 0.004, Tukey's test) (Fig. 3A–F). PSD95 expression was significantly increased at 12–24 h after HI than at 0–6 h (*P* = 0.029, Tukey's test) (Fig. 4A–F).

### Changes in synaptic protein expression after the use of 2-PMPA

**Fig. 5:**
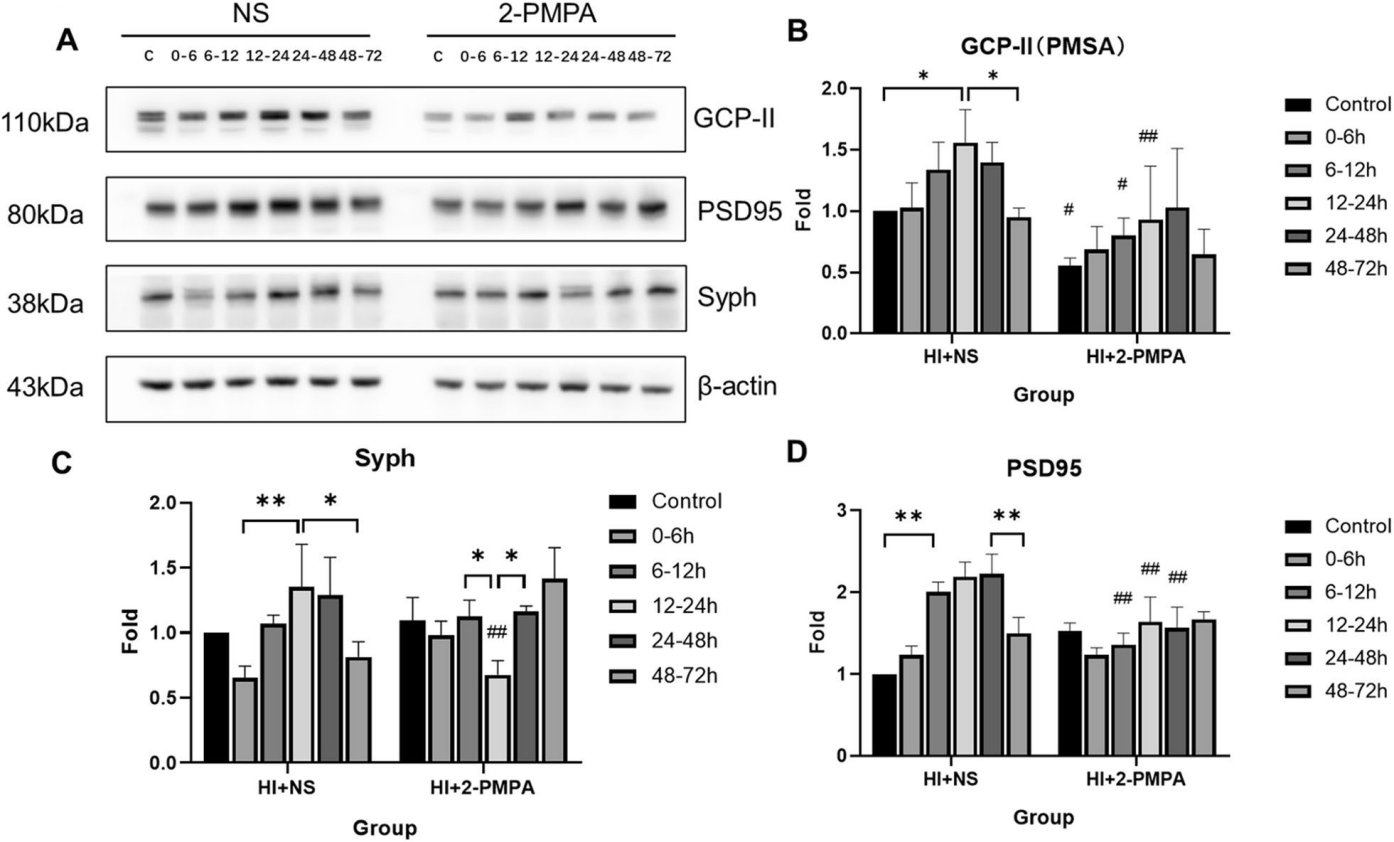
Changes in synaptic protein expression after the use of GCP-II inhibitor. **A** Changes in GCP-II, PSD95, syph and β-actin expression after the use of GCP-II inhibitor. **B**–**D** Bar graph of the expression changes of GCP-II, PSD95, and syph. (**P < 0.01, *P < 0.05, Turkey test; ^##^*P* < 0.01, ^#^*P* < 0.05, LSD test; data are expressed as mean ± standard deviation)

Synaptic protein changes after application of the 2-PMPA are shown in Fig. 5A–D. The GCP-II expression levels were significantly reduced in the control group, 6–12 h, 12–24 h of 2-PMPA group when compared with the corresponding NS group (*P* < 0.05, LSD test). At 12–24 h after HI, syph expression was significantly lower than in the 6–12 h, 24–48 h (*P* < 0.01, Tukey test) and NS group (*P* < 0.001, LSD test). Compared with the corresponding NS group, PSD95 expression levels were significantly reduced in the 6–12 h, 12–24 h, and 24–48 h of 2-PMPA group (*P* < 0.05, LSD test).

### Synaptical structural changes after HI damage and application of 2-PMPA

**Fig. 6:**
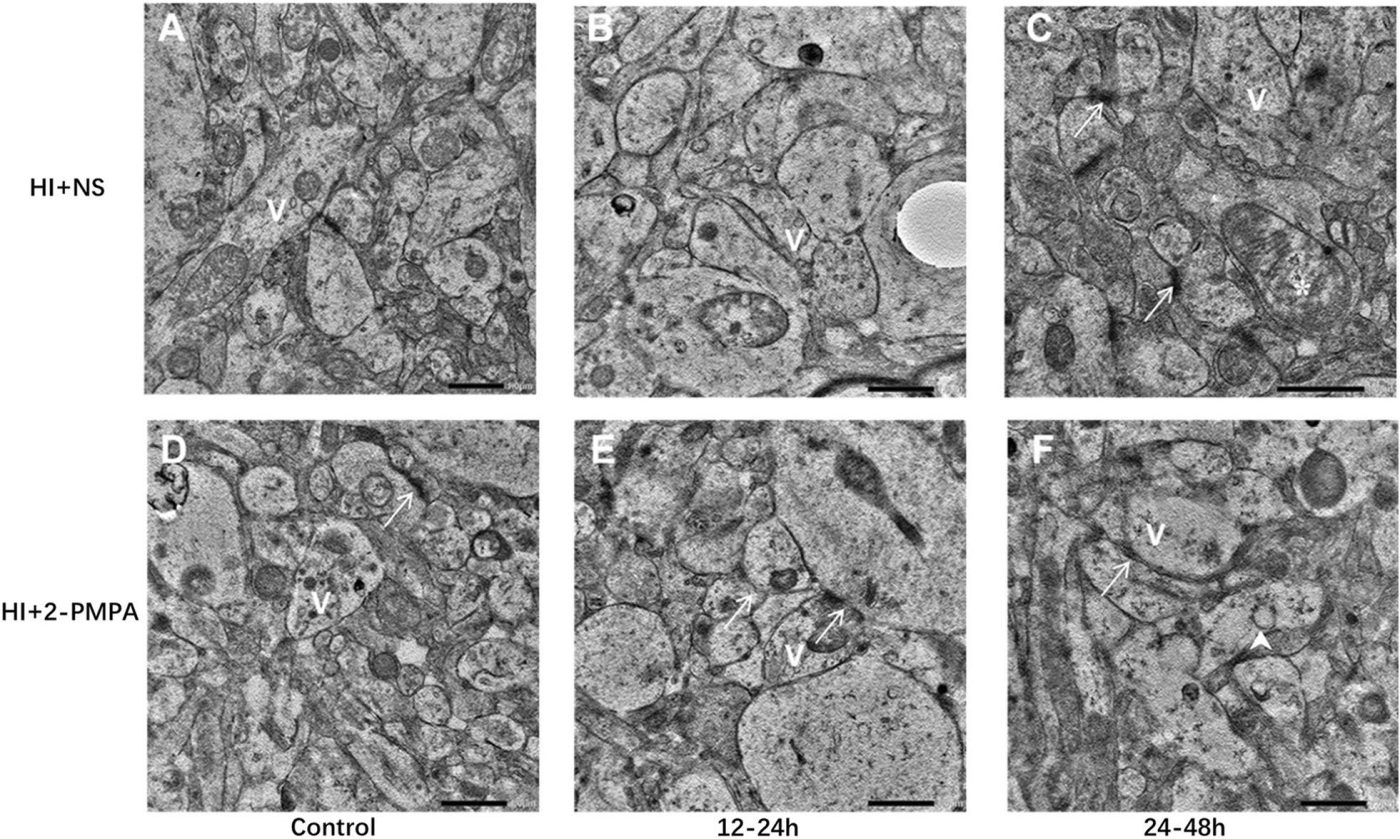
TEM observations of synaptic structural changes after HI damage and application with GCP-II inhibitor. The control group (**A** and **D**) are mainly composed of immature synapses, including neurotubules and small number of vesicles in the synapses, showing normal mitochondria. The number of synaptic vesicles (V) at 12–24 h (**B**) and 24–48 h (**C**) increased compared with control group (**A**). The synaptic swellings with discontinuous synaptic membrane structure; mitochondrial swelling at 24–48 h and mitochondrial crest fracture (*) after HI, besides, coated vesicle (uparrow) can be seen (**C**). Compared with the corresponding HI + NS group, the number of synaptic vesicles decreased at 12–24 h (**E**), 24–48 h (**F**) after HI in the 2-PMPA group. PSD structure (arrows) disruption (**E**) and thinning (**F**) are as indicated. Bar: 1 μm

The changes in synaptic structure in different time groups after HI injury and GCP-II inhibitor application are shown in Fig. 6A–F. The number of SVs increased at 12–24 h (Fig. 6B) and 24–48 h (Fig. 6C) when compared with control group (Fig. 6A) after HI. Post-HI the number of SVs decreased at 12–24 h (Fig. 6E) and 24–48 h (Fig. 6F) after the application of GCP-II inhibitors, as compared to the control group (Fig. 6D) and corresponding NS groups (Fig. 6B, C).

## Discussion

Synapses adapt to changes in the brain microenvironment by modifying their structure and strength following HI damage through a process known as "synaptic plasticity," which includes two mechanisms: homeostatic plasticity and Hebbian plasticity [31–33]. Hebbian plasticity is a kind of positive feedback mechanism that involves both long-term potentiation (LTP) and long-term depression (LTD) of synapses [34, 35]. Increased neuroplasticity in the developing brain is generally accepted to be beneficial for neurodevelopment, but excessive enhancement of excitatory circuits in pathological states like HI leads to adverse outcomes such as spontaneous seizures [36], suggesting that this positive feedback regulation may also be accompanied by damage. Homeostatic synaptic plasticity is a negative feedback mechanism in which neurons adjust synaptic strength by regulating presynaptic neurotransmitter release and the expression of presynaptic and postsynaptic receptors to maintain neurotransmitter homeostasis as well as counteract excessive excitation or inhibition [35, 37]. In this study, to investigate the regulation of synaptic plasticity after HI injury, we used ^1^H-MRS imaging to observe the dynamic changes of neurotransmitters NAAG and Glu after HI injury and analyzed the changes of metabotropic glutamate receptors and synaptic protein expression.

Neuronal activity in the mammalian cerebral cortex depends both on neurotransmitter transmission at the synapse and on the regulation of neuropeptides. In addition, neuropeptides are essential for the maintenance of synaptic network function [34, 38]. NAAG is able to specifically bind mGluR3 receptors on presynaptic membranes and astrocytes after synthesis in neurons. mGluR3 belongs to a group of G protein-coupled receptors that are coupled to inhibitory Gi / Go proteins. Activated mGluR3 inhibits adenylate cyclase activity, reduces cyclic adenosine monophosphate (cAMP) and cyclic guanosine monophosphate (cGMP) formation, and is able to inhibit voltage-sensitive Ca^2+^ channels [39–42]. Herein, we found that NAAG was negatively correlated with Glu at 0–12 h after HI, which indicating NAAG transformed with Glu. When NAAG levels increased 12–24 h after HI, so did mGluR3 expression, and NAAG was positively correlated with mGluR3, implying that NAAG binding to mGluR3 increased and downstream molecular pathways were activated during this period. The main function of voltage-sensitive Ca^2+^ channels is to control Ca^2+^ entry into the cell to influence the vesicular neurotransmitter release and to bind to and regulate Ca^2+^-dependent neurotransmitter release from the cytoplasmic domain of the transmembrane polypeptide of syph [43, 44]. Therefore, this study further confirmed the effect of NAAG on syph and SVs. At 12–24 h after HI, NAAG content increased with less modifications to syph expression levels, while syph expression was significantly reduced and TEM revealed a significant decrease in the number of SVs in the GCP-II inhibitory group. This lower modification to syph expression is perhaps due to the low NAAG content in the brain under physiological conditions and its changes are not enough to cause alteration in syph expression levels. While inhibiting GCP-II, NAAG hydrolysis is decreased, which can play a stronger function of presynaptic inhibition, inhibiting the voltage-sensitive Ca^2+^ channel and causing a reduction of Ca^2+^ influx, reduction of Glu vesicle release, and affecting Ca^2+^ binding to syph, thus reducing syph expression and number of SVs.

Signaling of NAAG-mGluR3 after HI affects synaptic plasticity by regulating the expression of postsynaptic proteins. PSD95 is one of the important skeletal proteins located at the postsynaptic membrane, affecting neurotransmitter delivery and ion homeostasis by directly acting on the NMDAR and promoting its stabilization and polymerization [29]. NMDAR is coupled to Ca^2+^ channels, and under pathological conditions such as HI, receptor overactivation promotes Ca^2+^ influx, which will lead to intracellular Ca^2+^ overload and cause postsynaptic cellular damage [45, 46]. This study identified that the expression levels of PSD95 were significantly lower after applying GCP-II inhibitors than in the NS group. Using TEM, we observed a disruption and reduced thickness in PSD (Fig. 6E, F), indicating that under the active state of NAAG-mGluR3 pathway, the decrease of glutamate release from SVs will lead to reduced glutamate binding to postsynaptic membrane and reduced PSD95 expression. This further inhibited NMDAR activation and lightened the excitotoxicity caused by Glu. In addition, this study also found that the coated vesicle structure appeared at 24-48 h after HI in the 2-PMPA group. Previous studies showed that this structure is one of the characteristics of synaptic membrane remodeling, associated with synaptic induction of LTP and PSD interruption [47], but the mechanism behind NAAG’s activity on this structure and the impact on long-term synaptic plasticity need to be further verified.

Post-HI NAAG-mGluR3 signaling may also further influence synaptic plasticity by regulating the expression of postsynaptic glutamate receptors. The mGluRs are involved in mediating electrical synaptic plasticity and neural circuit formation during brain development [48], mGluR5 also mediates synaptic excitotoxic signaling [49]. In the present study, mGluR3 was significantly negatively correlated with mGluR5 at 6–48 h after HI, indicating that mGluR3 was mutually regulated with mGluR5. During this period, we found that at 12–24 h after HI, NAAG content and mGluR3 expression were significantly increased and Glu was slightly decreased. Further, among the type I metabotropic glutamate receptors, mGluR5 expression were relatively decreased. These results indicated that mGluR5 activation was relatively reduced during this period, causing potentially diminished excitability of postsynaptic neurons. In contrast, 24–48 h after HI, NAAG content decreased while Glu content and expression of mGluR1 and mGluR5 increased, Glu was positively correlated with mGluR1/5. Further, TEM displayed mitochondrial swelling and mitochondrial crest cleavage (Fig. 6C), indicating that increased Glu release further causes enhanced postsynaptic excitatory signaling and causes cell damage. However, we also found that the reduction of mGluR5 at 12–24 h after HI was relative and not lower than the control group. The reason for this phenomenon may due to the low NAAG content and limited neuroprotective effect, and the influence of animal physiological changes [50, 51]. In view of this problem, we will further investigate the impact of NAAG on synaptic electrophysiology in the follow-up study.

## Conclusion

After 12–24 h of HI injury, we observed a one-time elevation in NAAG levels, which was consistent with the corresponding mGluR3 receptor expression trend. The NAAG maintains cortical synaptic plasticity and neurotransmitter homeostasis by inhibiting presynaptic glutamate vesicle release, regulating postsynaptic density proteins and postsynaptic receptor expression after pathway activation.

## Supplementary Information

**Additional file 1.** Animal modeling process.

## Data Availability

The datasets used and analysed during the current study are available from the corresponding author on reasonable request.

## Abbreviations

2-PMPA: 2-Phosphonomethyl pentanedioic acid
cAMP: Cyclic adenosine monophosphate
cGMP: Cyclic guanosine monophosphate
GCP-II: Glutamate carboxypeptidase II
Glu: Glutamic acid
HI: Hypoxic ischemia
iGluRs: Ionotropic glutamate receptors
LTD: Long-term depression
LTP: Long-term potentiation
mGluR3: Metabotropic glutamate receptor type3
mGluRs: Metabotropic glutamate receptors
NAA: N-acetyl-aspartate
NAAG: N-acetylaspartylglutamate
NAAGS: NAAG synthetase
NMDAR: NMDA receptor
NS: Normal saline
OD: Optical density
PSD: Postsynaptic density protein
SVs: Synaptic vesicles
syph: Synaptophysin
TEM: Transmission electron microscopy
