# Supplementary material for: The regulatory role of NAAG-mGluR3 signaling on cortical synaptic plasticity after hypoxic ischemia
Source: Cell Commun Signal. 2022 Apr 20;20:55. doi: 10.1186/s12964-022-00866-8 (PMC9022257; doi:10.1186/s12964-022-00866-8)
Supplement: Supplementary file 2 — Additional file 1. Animal modeling process. [file 12964_2022_866_MOESM2_ESM.docx]

**Additional file 1. Animal modeling process**

Anesthesia and mechanical ventilation: Anesthesia was administered using Sumianxin (Changchun Institute of Military Medical Research, Changchun, China) injected intramuscularly at a dose of 0.6 ml/kg. After the animal’s corneal reflex disappeared, tracheal intubation (diameter 2.5 mm) and ventilator-assisted breathing (U-25T bi-level airway pressure ventilator; Tianjin Yihejiaye Medical Technology Co., Ltd., China) was conducted with the respiratory rate set at 10 times/min and the pressure at 15–25 hPa. A Heal Force pulse oximeter (Shenzhen Hexin Zhondian Medical Equipment Co., Ltd., China) was used to monitor vital sign (pulse and blood oxygen saturation). An indwelling trocar in the right ear vein served as the venous channel.

Common carotid artery dissection: Skin was disinfected three times with iodophor followed by a median cervical incision, blunt dissection was performed in layers, and the common carotid artery was dissociated, with special attention paid to avoid damage to the carotid sinus and vagus nerve during the operation.

HI injury: The bilateral common carotid arteries were located by surgical sutures and occluded by artery clamps. Pigs mechanically inhaled a mixture of 6% oxygen and 94% nitrogen (Dalian Special Gas, Dalian, China) at the same time. After 40 minutes, the artery clamps were removed, 100% oxygen was resumed, and the cervical incision was sutured. The ventilator was then stopped and the tracheal tube was removed after spontaneous breathing resumed.

The animals in the control group underwent preoperative anesthesia and common carotid artery dissociation, while those in the experimental group received all surgical procedures including HI. The room temperature was kept at 28–30°C during the operation.
